# Mosquitoes are not the major culprits for the high burden of malaria in Nigeria: a commentary

**DOI:** 10.11604/pamj.2020.35.11.16972

**Published:** 2020-01-14

**Authors:** Celina Onyawoibi Aju-Ameh

**Affiliations:** 1 Department of Zoology, University of Jos, Jos, Plateau State, Nigeria

**Keywords:** Malaria, mosquitoes, Nigeria

## Abstract

Globally, Nigeria contributes the greatest proportion of the malaria disease burden. She currently bears the heaviest malaria burden (25% cases) and (19% deaths). Malaria is caused by *Plasmodium* parasites transmitted by female Anopheles mosquitoes however, a higher parasite biomass (99%) is found in man while only one (1%) is found in mosquitoes. Lending credence to this is the outcome of investigations carried out in Gboko and Otukpo Local Government Areas (LGAs); in which more humans (36.8%) had the malaria parasites than the anthropophagic female *Anopheles* (0.5%). Control efforts focused on mosquitoes are undermined by the actions or inactions of humans. Nigeria needs to self-audit her role in sustaining the heaviest burden of a preventable, curable disease that can also be eliminated. She can only ignore this imperative at her own peril.

## Commentary

Human malaria is an ancient disease that is preventable and curable but, can be fatal if not accurately diagnosed and properly managed. Malaria is caused by Plasmodium parasites transmitted only by female *Anopheles* mosquitoes. It is a foremost parasitic disease that has continued to be of public health importance and presently a global health priority. According to the World Health Organization (WHO)'s 2018 World Malaria Report [1], the WHO African Region had the highest (200 million or 92%) malaria cases in 2017, followed by the WHO South-East Asia Region with 5% of the cases and the WHO Eastern Mediterranean Region with 2%. A significant percentage (80%) of the global malaria burden was borne by fifteen countries in sub-Saharan Africa and India. Five countries accounted for almost half of all malaria cases worldwide: Nigeria (25%), Democratic Republic of the Congo (1%), Mozambique (5%), India (4%) and Uganda (4%) [1]. Several countries have successfully eliminated malaria in decades past from different regions of the world and records now show countries recently certified as malaria-free alongside future elimination targets [2]. Considerable progress must be made at reducing cases and deaths due to malaria in Nigeria for marked impact on national and international targets. This progress must be made by Nigerians for Nigerians. This article highlights some impediments to bending the malaria curve within the borders of Nigeria. These impeding factors must of a necessity be eliminated in order to eliminate malaria.

Malaria disease is sustained by the complex relationship between the triad: mosquitoes, parasite and man. The prevalence of the disease may be hinged on human actions and or inactions. To begin with, a higher parasite biomass (99%) is found in man while only one (1%) is found in mosquitoes [3]. In malaria heartlands, a larger segment of the human population is represented by the “infected” but asymptomatic population [3], and this is the existing phenomenon in most scenario. These symptomless groups are the inadvertent parasite reservoirs responsible for continued transmission. This position is further sustained by the outcome of investigations carried out in Gboko and Otukpo Local Government Areas (LGAs) in 2015. A cross sectional survey of 272 willing human subjects were screened for *Plasmodium* parasites using Rapid Diagnostic Test (RDT) and Microscopy (Table 1). The results (RDT-22.8%; Microscopy-36.8%) showed more humans than the mosquitoes had the parasites. Two hundred and fourteen female *Anopheles* mosquitoes engorged with human blood meal were tested for Plasmodium circumsporozoite antigen using Enzyme Linked Immunosorbent Assay (ELISA). Only one (0.5%) tested positive to the malaria parasite (Table 2). Mosquitoes ingest the parasite along with a blood meal from humans. The accompanying question is, who is infecting who-the mosquitoes or the man? Much as this one infective female *Anopheles* compromised with *Plasmodium* parasite (Table 2), can put the entire human population in the study community at risk of malaria disease; should the mosquito blood meal source be parasite free -a billion mosquitoes would not become infected. Only absence of malaria parasites equates zero transmission and ultimately zero malaria. This article does not stand to completely acquit the mosquitoes but seeks to bring to bear some non-mosquito undercurrents that may be responsible for the high burden of malaria in Nigeria. Continued neglect of these salient challenges may render the malaria control effort and ultimately elimination a mirage. Attaining a zero-malaria status in Nigeria is possible but these impeding factors of a necessity must be urgently addressed. In a broad sense some of these factors are: Leadership, Integrity, Infrastructure deficiency and behavioral challenge to mention a few. Narrowing it down to malaria we have elitist decadence and poverty of the mind. Taking a brief look at these afore mentioned factors:

**Table 1:** malaria parasite infection rate in asymptomatic subjects within Gboko and Otukpo LGA, Benue, State Nigeria

Settlement	No Examined	RDT(%)	MicroT(%)	RDT&MicroT (%)	PCR(%)
**Rural**	238	59(24.8)	95(39.9)	32(13.4)	2(0.8)
**Urban**	34	3(8.8)	3(8.8)	1(2.9)	
**TOTAL**	272	62(22.8)	98(36.8)	33(12.1)	2(0.7)
**P-value**		0.003	0.001	0.078	0.826

NS=No Significant difference in Columns where P > 0.05; RDT-Rapid diagnostic test; MicroT-Microscopy Test; PCR-Polymerase Chain Reaction; Only willing participants were screened. PCR was carried out for only the first 15 enrollee

**Table 2:** sporozoite rate of female *Anopheles* species between urban and rural sites in Gboko and Otukpo local government areas

Sites	No analysed	No infected	No uninfected
**Rural**	204.0	1.0	203.0
**Urban**	10.0	0.0	10.0
T**OTAL**	**214.0**	**1.0**	213.0
T**=0.823**		Df=1	P=1.00

### Leadership challenge

Siollum [4], put it succinctly when he said, when it gained independence from the UK in 1960, hopes were high that, with mineral wealth and the most educated workforce in Africa, Nigeria would become Africa's first superpower and a stabilizing democratic influence in the region. However, these lofty hopes were soon dashed, and the country lumbered from crisis to crisis with the democratic government eventually being overthrown in a violent military coup in January 1966". She has since continued indeed from crisis to crisis and now enmeshed in monumental crises driven by politics, religion and ethnicity. Unfortunately, the mosquitoes and malaria are not limited by any of these-the war against an advanced disease like malaria cannot be won amidst a cesspool of multilayered internal wars. In this context, she now needs a leadership team that is focused, selfless and stable to get her out of the woods to the plinth of global relevance. Barack Obama on leadership in America said, “I think about America and those who built it. This nation's founder, who somehow rose above petty ambitions and narrow calculations to imagine a nation unfurling across a continent. And those like Lincoln and King, who ultimately laid down their lives in the service of perfecting an imperfect union” [5]. Taking a cue from the above, national interest must cease to be mortgaged for personal interest. Ours may be an imperfect union but there is strength in diversity. Both the leadership and followership must orientate towards doing the right things, at the right time and in the right way to set the nation back on course again.

### Integrity

The hydra headed monster, corruption is woven into the very fabric that gives Nigeria life and rife among majority of the leaders, swamping down to the least. Wrong values and priorities are also a signature among most of us exemplified by government officials and the elite [6]. As the momentum for malaria elimination steams up, one sector that corruption must not be allowed to permeate is health. Integrity and transparency in data collection/collation; dispensing of donated malaria commodities and judicious use of funds is fundamental to bending the malaria curve in Nigeria. Muddling up data, diverting funds or misappropriating scarce resources is fraudulent and could mean death for vulnerable risk groups. Soyinka putting it vividly: “A liar will invariably be a fraud, a rogue, a murderer” [7]. While the elite can access health care outside the country, the poor people are left to pay the price of corruption not only in the health sector but in every sphere of life so affected. In the Audacity of Hope, Barack Obama said: I'm reminded that the actions of those in power have enormous consequences-a price that they themselves almost never have to [5]. There is a dire need to raise the bar of our integrity index in all spheres of endeavor. Shouldn't we dare to address the dire need to raise the bar of our integrity index in all spheres of endeavor?

### Infrastructure deficiency

In the 50s, much of the work towards malaria eradication was done on foot, by boat, donkey, horse or camel back where vehicles could not access [8]. A similar modus operandi may play out still in Nigeria if total coverage is to be attained for some of the malaria control tools. Infrastructure deficiency ranges from: inaccessible roads to vulnerable risk communities, absence or underperforming health systems, inadequate health centres and non-performing regulatory bodies. Capping these are primitive housing structures that offer no protection but rather aid malaria vector invasion into human habitation. Besides the physical infrastructure deficiency there is the challenge of clueless employees and unskilled personnel (inadequate skilled human resource) resulting directly from the abysmal educational sector. Addressing the Nigerian nation at the National Economic Council in Abuja, Bill Gates said, “People without roads, ports, and factories can't flourish. And roads, ports and factories without skilled workers to build and manage them can't sustain an economy. I urge you to apply this thinking to all your investments in your people” [9]. The impoverish cannot flourish and poverty is a predisposing factor for malaria disease. Compounding Nigeria's malaria plight is her free fall into extreme poverty. Of the five countries responsible for almost half of all malaria cases worldwide, these two countries: Nigeria (25%) and Democratic Republic of the Congo (11%) accounts for the highest malaria burden [1]. Both countries are also responsible for the highest number of people entering into poverty per minute [10].

### Behavioural challenge

Behavioural Challenges that aid in sustaining the malaria scourge are inclusive of but not limited to: neglect of sanitary measures, poor routine hospital data collection culture, poverty of the mind, elitist decadence, and creation of breeding places close to residential areas. Furthermore, humans are responsible for adulterated drugs and insecticides as well as their miss-use. They are responsible for poor uptake of control interventions, unethical use of mosquito bed nets or non-use. A compromised female anthropophagic *Anopheles* malaria vector is a big deal but the animal most culpable presently in Nigeria for the proliferation and status of this advanced disease is not the arthropod but the homo sapiens. Can Nigeria with international support and a strong political will, beat malaria? The answer is definitely a yes however, behavioral, social, environmental and economic changes by her citizens will be needed to make a head way in the fight against malaria. When the rest of the world is done getting rid of malaria, Nigeria should not stand as a threat and a parasite source for both mosquitoes and humans since the world is now a global village. These changes are imperative to changing Nigeria's malaria narrative.

**Box 1: poverty of the mind:** a situation where the victim is asking to be paid before control intervention can be deployed or outright rejection of interventions or misuse even after explanations on advantages of use and dire consequences of non-use/misuse have been given.

**Elitist decadence:** a situation where those in positions enabled by knowledge and power use same for self-aggrandisement by diverting funds, selling commodities/services meant for free and falsification of reports and data to justify such.

**Recommendation:** Nigeria needs to take the plethora of statistics on its numerous failures and begin to systematically proffer solutions alongside an efficient implementation machinery. Leadership needs to reduce the overwhelming burden of malaria on the Nigerian citizen within her boarders. Charity is said to begin at home- it is time wealthy and influential Nigerians begin to pour funds into research and combating endemic diseases in their villages.

## Conclusion

Mosquitoes cannot get rid of malaria, humans can get rid of malaria. We must re-set our priorities as a nation, pursue and eliminate malaria from within our boarders. Zero malaria begins with each Nigerian citizen.

## Competing interests

The author declares no competing interests.
